# Supplementary material for: Tracing the origin of lithium in Li-ion batteries using lithium isotopes
Source: Nat Commun. 2022 Jul 26;13:4172. doi: 10.1038/s41467-022-31850-y (PMC9325982; doi:10.1038/s41467-022-31850-y)
Supplement: Supplementary file 1 — Supplementary Information [file 41467_2022_31850_MOESM1_ESM.pdf]

Supplementary Information for tracing the origin of lithium in Li-ion batteries using lithium isotopes

***Table 1** - **Summary of analytical data and description of the samples analysed in this study**.*

The number of measurements marked with an asterisk (*) indicates that different preparations were applied to the sample (dissolution and/or chemical purification).

Name	Type	supplier/lot n°.	Deposit	Country of origin	δ^7Li	±2σ	Number of measurements
Li 20	spodumene concentrate	Keliber	Finland	Finland	1.1	0.3	4*
Li 21	spodumene concentrate	North American Lithium	La Corne Quebec	Canada	3.1	0.3	4*
Li 28	spodumene concentrate	Sayona	Authier Quebec	Canada	4.4	0.4	2
Li 29	β-spodumene	Keliber	Finland	Finland	1.1	0.4	3
Li 30	Analcime	Keliber	Finland	Finland	-0.9	0.1	3
Li 01	lithium hydroxide monohydrate battery grade	Alfa Aeser/ 10225937		Russia	2.7	0.4	6
Li 02	lithium hydroxide monohydrate ≥98%	Acros Organics/ A041350		Russia	12.2	0.1	3
Li 03	lithium hydroxide monohydrate ≥98.5%	Fluka/ I2050		USA	13.1	0.3	3
Li 04	lithium hydroxide monohydrate ≥99.0%	Sigma Aldrich/ BCBW3215		USA	8.1	0.1	2
Li 27	lithium hydroxide monohydrate ≥99.0%	Sigma Aldrich/ BCBV9203		USA	8.5	0.2	2
Li 05	lithium hydroxide monohydrate 98.5-101.5%	Sigma Aldrich/ SLCC6671		UK	14.8	0.4	4
Li 06	lithium hydroxide monohydrate extra pure	Fisher Chemical/ 1737794		UK	13.8	0.4	4
Li 22	lithium hydroxide monohydrate battery grade	Tianqi Lithium		China	7.5	0.2	2*
Li 07	lithium hydroxide monohydrate battery grade	Leverton/ 12640	Salar Atacama Chile	UK	12.7	0.4	26
Li17	lithium hydroxide monohydrate battery grade	SQM/ 70149	Salar Atacama Chile	Russia	13.7	0.3	5
Li26	lithium hydroxide monohydrate battery grade	Keliber	Finland	Finland	6.6	0.4	8*
Li 13	lithium carbonate extra pur	Fisher Chemical/ 1988560		UK	2.1	0.5	25
Li 12	lithium carbonate >99%	Acros Organics / A0415714		India	12.4	0.1	3
Li 09	lithium carbonate ≥99.0%	Sigma Aldrich/ WXBD3215V		China	10.0	0.3	3
Li 19	lithium carbonate battery grade	Ganfeng Lithium		China	5.1	0.5	5
Li 23	lithium carbonate battery grade	Tianqi Lithium		China	7.8	0.5	3

Li18	lithium carbonate battery grade	Leverton/12714	Salar Atacama Chile	UK	11.9	0.4	5
Li 25	lithium carbonate battery grade	SQM/ 1120080509	Salar Atacama Chile	Chile	13.3	0.1	2
Li 11	lithium carbonate 99%	Alfa Aesar/ W07E034		Argentina	7.4	0.5	4
X	active material NMC333	TOB (factory A)		Fujian, China	10.6	0.2	3*
Y	active material NMC532	TOB (factory A)		Fujian, China	8.3	0.5	3*
Z	active material NMC622	TOB (factory B)		Fujian, China	0.6	0.1	3*
W	active material NMC811	TOB (factory C)		Fujian, China	11.6	0.4	3
A1c	Cathode sheet A	unknown		South Korea	10.3	0.6	4*
A8c	Cathode sheet A	unknown		South Korea	10.4	0.6	2
B1c	Cathode sheet B	unknown		South Korea	10.0	0.4	5*
B6c	Cathode sheet B	unknown		South Korea	10.5	0.6	2
C1c	Cathode sheet C	unknown		South Korea	10.5	0.6	2
C6c	Cathode sheet C	unknown		South Korea	10.2	0.6	4*
D1c	Cathode sheet D	unknown		South Korea	10.7	0.6	2
D6c	Cathode sheet D	unknown		South Korea	10.3	0.7	5*
A7	Cathode sheet A	unknown		South Korea	10.6	0.5	2
B3	Cathode sheet B	unknown		South Korea	10.4	1.1	2

***Table 2** - Summary of analytical data and description of the samples synthetised in this study.*

**Name**	**Type**	**Salt used for the synthesis**			**δ^7Li**	**±2σ**	**n**
		**Name/type**	**Supplier**	**Country of origin**			
Li01 NMC622	active material NMC622	Li01/ Li hydroxide	Alfa Aeser	Russia	3.2	0.2	2
Li01 NMC811	active material NMC811	Li01/ Li hydroxide	Alfa Aeser	Russia	3.0	0.5	3
Li13 NMC622	active material NMC622	Li13/Li carbonate	Fisher Chemical	UK	2.0	0.6	2
Li13 NMC811	active material NMC811	Li13/Li carbonate	Fisher Chemical	UK	2.1	0.5	2
Li17 NMC622	active material NMC622	Li17/Li hydroxide	SQM	Russia	13.8	0.5	3
Li17 NMC811	active material NMC811	Li17/Li hydroxide	SQM	Russia	13.9	0.3	2
Li18 NMC622	active material NMC622	Li18/Li carbonate	Leverton	UK	12.0	0.3	2
Li18 NMC811	active material NMC811	Li18/Li carbonate	Leverton	UK	11.9	0.5	3

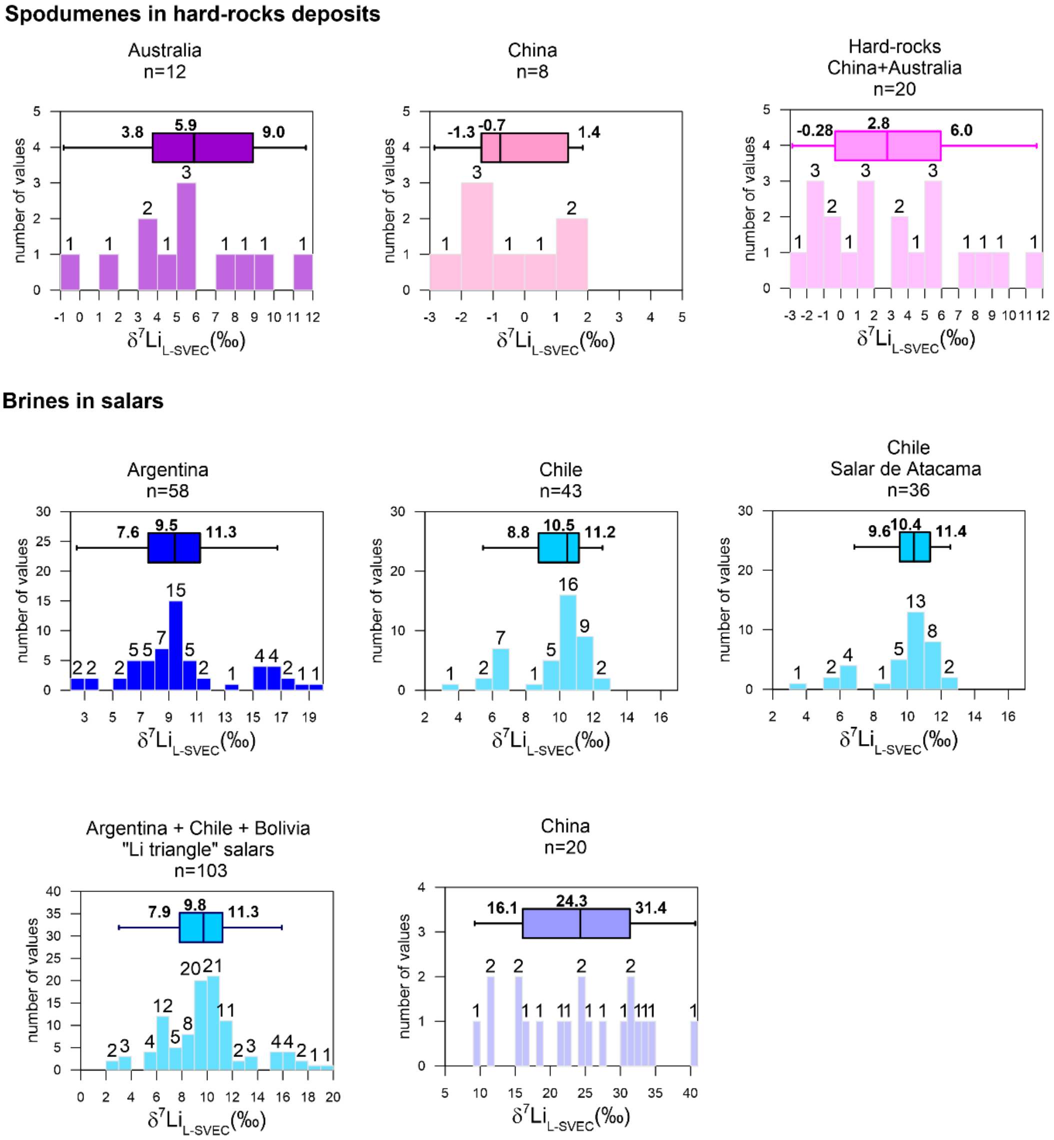

Fig. 1 - *Distribution of Li isotope compositions for natural samples.*

Spodumenes from Australia (n=12) and China (n=8), and brines from Argentina (n=58), Chile (n=43), Chile (Salar de Atacama, n=36), "Li triangle" salars (n=103) and China (n=20).

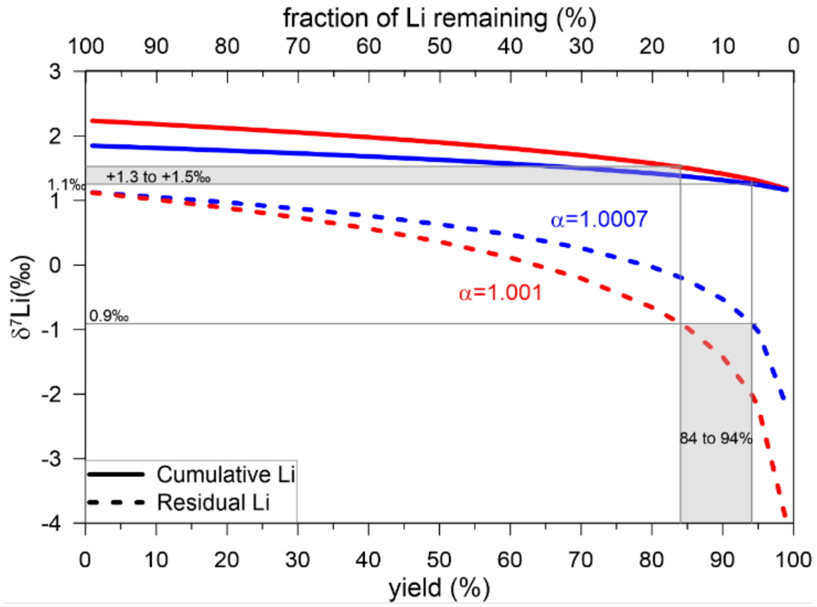

Fig. 2 - ***Rayleigh fractionation diagram simulating residue and produced compositions of Li during leaching****.*

For a starting composition of δ⁷Li in ores of +1.1‰, the δ⁷Li of analcime of -0.9‰ and a lithium leaching extraction yield given by Keliber between 84% and 94%. Kinetic fractionation factors α are estimated 1.0007 and 1.001, and δ⁷Li values are estimated between +1.3 and +1.5 ‰ for Li in solution.

Note:

Laboratory experiments for estimating fractionation due to purification by cation exchange resin

Experiments 1 and 2 described below were performed in a clean room to avoid contamination.

Experiment 1. Five aliquots of a Li-13 sample volume corresponding to 100 ng Li were dried on a hot plate (samples 1, 2, 3, 4 and 5). The residues were diluted in 0.2 M HCl and loaded on five cation-exchange columns (columns 1, 2, 3, 4 and 5) filled with 2.6 mL AG 50W−X12 resin, composed of sulphonic acid functional groups attached to a styrene divinylbenzene copolymer lattice (Fig. 3). Different volumes of 0.2 M HCl (12 mL, 13 mL, 14 mL, 15 mL and 21 mL) were used for eluating the Li in each column, to obtain various Li recoveries. The eluates were collected in beakers, dried on a hot plate, and then re-dissolved in 0.5 M HNO_3. Li recovery for each eluate volume was determined by analysing the Li contained in each aliquot after chemical separation. The Li content of each sample was determined by ICP-MS in the BRGM laboratories.

Experiment 2 was performed like experiment 1, except that the eluates obtained were dedicated to the analysis of δ^7Li values by MC-ICP-MS at BRGM.

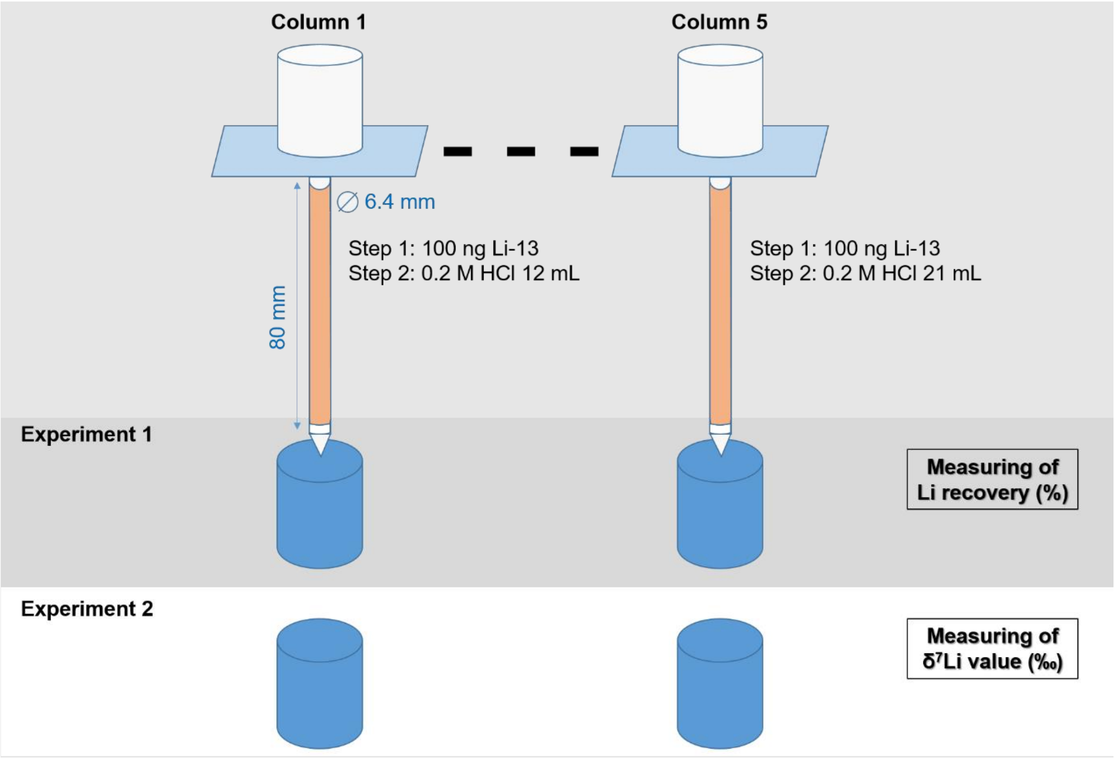

Fig. 3 - ***Laboratory experiments 1 and 2***.

These experiments served to estimate the fractionation factor due to purification by cation exchange resin; they involved 5 columns, but only columns 1 and 5 are shown.

The Li recovery results for each volume of elution and the associated δ^7Li values of the eluate are shown on Figure 4. Using a Rayleigh model, we can estimate the kinetic fractionation factor α. Due to incomplete Li recovery of during the cation exchange purification, this was estimated to be 1.055. These experiments showed that even a 95% Li yield leads to high fractionation between Li+ and purified Li+ (ΔLi $_{\text{purified Li+-Li+}}$ >+8‰).

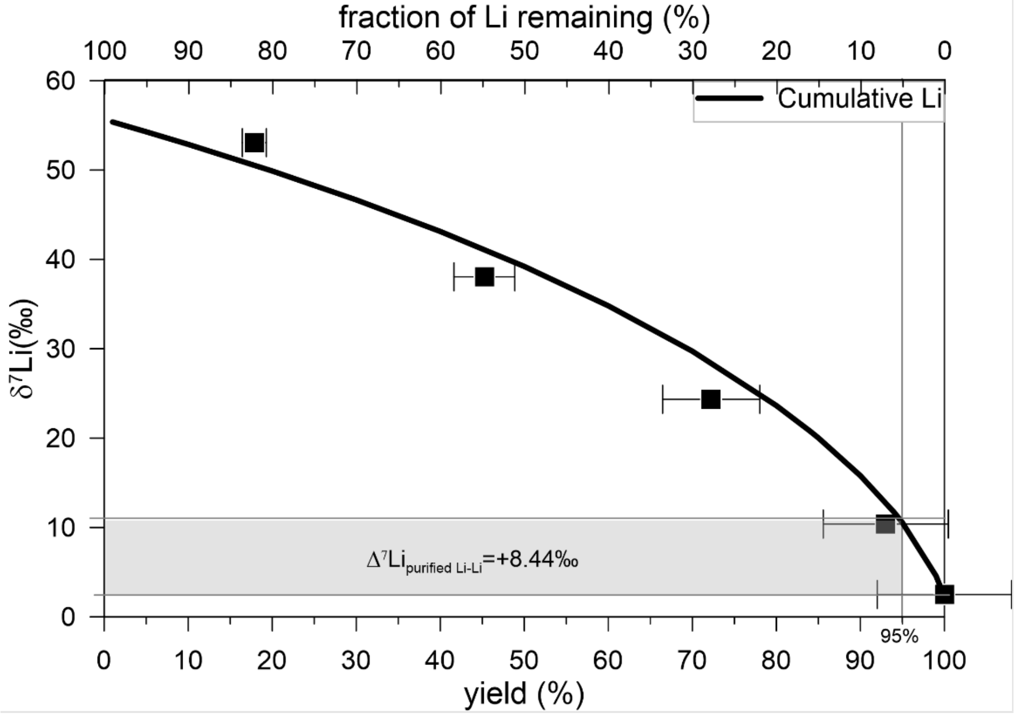

Fig. 4 - *δ⁷Li values of eluate* versus *Li recovery for each volume of elution used in experiments 1 and 2.*

The volumes to purify Li by cation exchange resin were 12 mL, 13 mL, 14 mL, 15 mL and 21 mL. The black line represents the Rayleigh fractionation diagram simulating the produced compositions of Li during purification for a kinetic fractionation factor α of 1.055.
